# Diphtherite

**Published:** 1860-07

**Authors:** J. H. Barbour

**Affiliations:** Falmouth, Ky.


### ARTICLE XIII.

*Diphtherite.* By J. H. BARBOUR, M. D., Falmouth, Ky.

THIS disease first made its appearance in this county in the spring of 1857 in epidemic form, and has been prevailing in different portions of the county ever since. Upon its first occurrence, it was a very fatal disease, and although still dangerous and fatal, it seems to have lost somewhat of its virulence. It made its first appearance in the lower portion of this county, during an epidemic of scarlet fever, and was generally recognized by physicians as being a form of that disease, and is in fact, yet so considered by some.

It did not present itself in my practice until the fall of 1857, and then also during the worst epidemic of scarlatina I have ever witnessed. I immediately recognized the destructive features of this disease. In my experience, patients that have had the scarlet fever, enjoy no immunity from diphtherite ; nor do diphtherite patients enjoy any immunity from scarlet fever. During the last two years and a half it has fallen to my lot to treat a great number of patients, of all ages, sex and color, affected with this disease.

Diphtherite often commences with a chill, followed by fever, external swelling of the throat, painful and difficult deglutition, etc. In other cases the fever is intermittent, without chill, and with sickness of the stomach—the fever lasting three or four hours each day, and from three to five days before the throat symptoms are manifest, the patient appearing quite well, excepting during the fever.

In many cases the disease comes on in a more insidious manner: the patient complains but little, although it is evident from the paleness and lassitude that something is wrong ; the surface is moist, cool and pale ; the pulse remarkably frequent, (this frequency of the pulse I have found to be one of the earliest symptoms, indicating the approach of the disease before other symptoms were set up.)

In some cases slight external swelling of the throat. Upon pressing down the tongue with a spoon, and examining the tonsils, if the case is in its early stage, you will find the tonsils and soft palate red and slightly swollen, with one or more small patches on the tonsils of a yellowish appearance, resembling a small piece or patch of buckskin spread upon the glands. If the disease continues to progress, this membrane extends until it covers all the visible portions of the throat when the tongue is depressed ; as the case progresses, this membrane may break down into an ugly, gray slough, or the false membrane be exfoliated entire.

In other cases the tonsils are covered with a grayish, ragged, sloughing surface from the first. And I have seen in two cases a smooth, milky appearance, as though the mucous membrane had been touched with a strong solution of nitrate of silver. In both of these cases the laryngial symptoms predominated from the first, and they both resulted fatally.

In some cases the tonsils are very much swollen, and a portion of the gland protruding from the base, causing it to resemble an acorn in its cup.

In bad cases I have seen the tonsils and soft palate assume a gangrenous look, and the diphtheritic membrane come of like bits of soft, thin buckskin, the soft parts under it offering no more resistance to the pencil of nitrate of silver than would tender cheese. These were fatal cases.

In a few cases I have had to contend against exhausting hemorrhage from the ulcerated surfaces of the throat.

In two cases, the sloughing of the soft palate, and the destruction of the surrounding parts has been such as to very much impair the power of deglutition ; for, upon attempting to swallow, a great portion of the food or fluid would be thrown out through the nasal passages. In these cases the voice was very much impaired, or lost for the time—owing, as I supposed, probably to the destruction of the soft palate ; yet, as both of these cases had laryngial symptoms, may it not have been owing in part to the disease extending into the larynx, and affecting or stiffening the chordæ vocales for a time ?—for both of the cases recovered, and in a few weeks the voice was entirely restored.

The external portions of the throat in most cases were but little swollen, but in some few cases they were much swollen and œdematous.

As to the fatal termination of this disease, I consider it owing to its extension into the larynx and trachea. I have seen no cases terminate fatally excepting such as where the laryngial symptoms were present, as shown by hoarseness, difficult and croupal respiration. In such cases, patients, unless quite young, could not be forced to keep their beds, but would sit up, or walk about the room. Little ones would get up and walk across the room to get a drink of water, up to the moment of death. A little boy of ten years old set up, and whittled a stick the most of the day, and in the evening put on his coat and drew on his boots, which were rather hard to draw, a few moments before he died. The difficulty of breathing was great during this time. He could not be induced for a moment to assume any other posture but the sitting or standing one.

I do not consider diphtherite contagious. If so, it is very uncertain in its action. I have seen those most exposed, entirely escape, while those in no manner exposed have the disease.

I have never been able in pure cases of diphtherite to detect any cutaneous eruption or rash.

In the beginning with us, diphtherite was associated with scarlatina; but not of late. It also in some cases was associated with intermittent fever, as in my own case, and also in other cases that I observed during the fall of 1858. I had had an attack of intermittent fever, and arrested it with quinine. In one month it returned with a severe chill, followed by a fever. I resorted to quinine. The chill did not return, but I discovered a dryness and soreness, with severe cutting pain, upon attempting to swallow. This led me to examine my throat, when I discovered a yellow patch upon my left tonsil about the size of a half dime. I treated it, and in three days it disappeared.

*Prophylaxis.*—I have used the chlorate of potassa, in solution, given internally as a preventive.

*Treatment.*—If there is much excitement in the beginning, I give an emetic of ipecac, and followed, if the bowels are constipated, with some mild cathartic, such as rhubarb. After the action of the emetic, if one is given, or otherwise immediately, I cauterize the diphtherite patches on the tonsils with nitrate of silver, applying the stick direct. I think it much neater than the solution, and can be applied where you wish it, and there only. I load the end of a quill with the stick—the best caustic holder I know of, and always at hand in a country practice. With this I cauterize the throat once every twenty-four hours, so long as necessary. I make a saturated solution of the chlorate of potassa, to which, if the case requires it, I add quinine and Bourbon whiskey.

℞ Chlorate of potassa, ℥j
Sulph. quinine, grs. xvj.
Water, ℥ xvj

Dose, from a teaspoonful to a tablespoonful every three hours, according to age. In cases where there is a pale, moist, cool skin, a frequent and soft pulse, the addition of the whiskey to the above formula will prove of great benefit.

As a stimulating gargle I use—

℞ Apple vinegar, ℥ viij.

Capsicum, ʒ j.

Muriate of soda, ʒ j. M.

Use three or four times a day as a gargle. As an astringent gargle, I use a decoction of oak or persimmon bark, cold.

As a styptic in hemorrhage from the ulcerated surface of the throat, I used a strong solution of the sulphate of zinc and ice.

The external application that I used in all my cases was a piece of old bacon bound to the throat. The bacon usually in from one to three days brings out a crop of pimples, producing a safe and valuable counter irritation. Owing to the tendency of diphtheritic inflammation to attack abraded surfaces, I do not consider it judicious to use the ordinary blistering preparations.

This course of treatment in my hands has been successful in every case but one that I saw before the laryngial symptoms were set up. It has been adopted by other physicians with like success.

As a constitutional remedy, I rely upon the chlorate of potassa and quinine, with the addition of a stimulus, when required.

As a topical application, I rely upon the nitrate of silver, with the gargles above mentioned. In many cases I allow a liberal fluid diet, if it can be taken.

[*Cincinnati Lancet and Obs.*

